# Pathological Evaluation of Porcine Circovirus 2d (PCV2d) Strain and Comparative Evaluation of PCV2d and PCV2b Inactivated Vaccines against PCV2d Infection in a Specific Pathogen-Free (SPF) Yucatan Miniature Pig Model

**DOI:** 10.3390/vaccines10091469

**Published:** 2022-09-05

**Authors:** Yun-Hee Noh, Seung-Chai Kim, Chang-Gi Jeong, Seung-Chul Lee, Dong-Uk Lee, In-Joong Yoon, Won-Il Kim

**Affiliations:** 1 Choong Ang Vaccine Laboratories Co., Ltd., Daejeon 34055, Korea; 2 College of Veterinary Medicine, Jeonbuk National University, Iksan 54596, Korea

**Keywords:** porcine circovirus type 2 (PCV2), Yucatan miniature pig, cross-protection, inactivated vaccine, PCV2b, PCV2d

## Abstract

Porcine circovirus type 2 (PCV2) is an economically important swine pathogen that causes porcine circovirus-associated diseases (PCVADs). The objective of this study was to evaluate the use of specific pathogen-free Yucatan miniature pigs (YMPs) as an experimental model for PCV2d challenge and vaccine assessment because PCV2-negative pigs are extremely rare in conventional swine herds in Korea. In the first experiment, every three pigs were subjected to PCV2d field isolate or mock challenge. During three weeks of experiments, the PCV2d infection group exhibited clinical outcomes of PCVAD with high viral loads, lymphoid depletion, and detection of PCV2d antigens in lymphoid organs by immunohistochemistry. In the second experiment, three groups of pigs were challenged with PCV2d after immunization for three weeks: a nonvaccinated group (three pigs), a PCV2b-Vac group vaccinated with a commercial PCV2b-based inactivated vaccine SuiShot^®^ Circo-ONE (five pigs), and a PCV2d-Vac group vaccinated with an experimental PCV2d-based inactivated vaccine (five pigs). During the three weeks of the challenge period, nonvaccinated pigs showed similar clinical outcomes to those observed in the PCV2d infection group from the first experiment. In contrast, both the PCV2b and PCV2d vaccinations produced good levels of protection against PCV2d challenge, as evidenced by reduced viral loads, improved growth performance, high virus-neutralizing antibody titers, and less development of PCV2-associated pathological lesions. Taken together, these data suggest that YMPs could be an alternative model for PCV2 challenge experiments, and these animals displayed typical clinical and pathological features and characteristics of protective immunity induced by the vaccines that were consistent with those resulting from PCV2 infections in conventional pigs.

## 1. Introduction

Porcine circovirus type 2 (PCV2), a small single-stranded circular DNA virus in the *Circoviridae* family, has been identified as one of the major swine pathogens of porcine circovirus-associated diseases (PCVADs), which often lead to high economic losses in the swine industry worldwide. After the initial description of postweaning multisystemic wasting syndrome (PMWS), enteric, respiratory, and reproductive disorders were subsequently linked with PCV2 under the name porcine circovirus diseases or PCVADs, including reproductive failure, porcine respiratory disease complex (PRDC), porcine dermatitis and nephropathy syndrome (PDNS), granulomatous enteritis, proliferative and necrotizing pneumonia (PNP), necrotizing lymphadenitis, and possibly exudative epidermitis [1,2,3]. Pigs with PCVAD are often observed to have enlarged lymph nodes and show decreased weight gain or wasting combined with many other nonspecific clinical signs, such as dyspnea, pallor, diarrhea, and jaundice [4]. In addition, high PCV2 viremia and viral load in tissues, granulomatous inflammation, lymphocyte depletion, and lymphoid system dysfunction causing immunosuppression have been identified as the hallmarks of severe PCV2 infection [5].

PCV2 is often classified as subtypes a, b, c, d, e, f, g, or h based on ORF2 sequence analysis [6]. Ongoing debate continues regarding the nomenclature of PCV2 genotypes. After PCV2 genotypes were identified, PCV2a was the primary genotype. However, in approximately 2003, a genetic shift occurred from PCV2a toward PCV2b [7]. Subsequently, a third genotype, PCV2c, was retrospectively reported in Denmark in the 1980s [8]. A fourth genotype, PCV2d, with an additional amino acid resulting from a mutation in the ORF2 stop codon, has also been recently described, although it had already been retrospectively detected in Switzerland in 1998 [9,10]. In fact, it was initially referred to as a mutant of PCV2b and was further linked to potential vaccination failure cases [11]. Further studies have shown that PCV2d has become prevalent globally. More recently, a new genotype (PCV2e) has been identified in swine samples from the United States and Mexico [12,13]. In addition, a Chinese study found novel viral sequences clustering differently from the existing ones; they were tentatively classified as genotypes PCV2f, PCV2g, and PCV2h [14]. In Korea, PCV2a was the most prevalent genotype from 1996 until the 2000s. However, after 2005, PCV2b became more widespread [15,16]. PCV2d was then identified in 2007, and many studies have reported that this genotype is increasing in prevalence and replacing the formerly prevalent PCV2a and PCV2b [6,16,17].

The first commercial vaccines were made available in 2004 in Europe and in 2006 in North America, and they have been widely received among pig farmers worldwide [5]. The decrease in morbidity and improved production efficiency that was observed after the adoption of the PCV2 vaccine unambiguously demonstrated the impact of PCV2 on the health of pigs [18]. Current commercial PCV2 vaccines are typically inactivated whole-virus or subunit vaccines based on the PCV2a genotype. However, despite the widespread use of the PCV2 vaccine, PCV2d has gradually replaced PCV2a and PCV2b, becoming the predominant global PCV2 strain. There are increasing concerns regarding immune escape due to the overuse of vaccines, as PCV2d has been found in several farms with PMWS-like clinical signs, such as growth retardation and histopathological lymphoid lesions, which are often associated with vaccine failure [10,19]. Recently, this concern led to the performance of diverse studies on cross-protection between commercialized vaccine genotypes and field genotypes [20,21]. Several previous studies in the USA showed that selected commercial subunit or chimeric PCV2 vaccines (PCV2a-based vaccines) can protect pigs against PCV2d challenge [11,22,23]. In Korea, the PCV2a and PCV2b vaccines are currently available on the market. Recent studies have shown that commercial PCV2a-based vaccines could provide protective immunity against Korean PCV2d strains [24]. However, there is currently no evaluation of whether the PCV2b-based vaccine defends against Korean PCV2d strains.

It has been suggested that it is difficult to experimentally reproduce clinical PCVAD that is similar to what is observed in pigs naturally infected with PCV2, and the presence of viral antigens in the absence of any symptoms of clinical disease has been difficult to explain in many experimental models, including cesarean-derived, colostrum-deprived (CDCD), colostrum-deprived snatch-farrowed, and conventional pigs [25,26]. Gnotobiotic (GN) pigs are widely used to study the direct effect of bacterial and/or viral infections on disease progression, as they provide an unbiased view of the pathogenesis associated with a single infectious agent [26]. Several studies have experimentally reproduced severe PMWS or PCVAD lesions using GN pigs with PCV2 single infection [26,27]. However, experimental studies using GN pigs are limited, as special gnotobiotic facilities and technical expertise are required for experimental infection [28,29]. Therefore, several miniature pig lines, such as the Yucatan, Göttingen, Sinclair, and Hanford lines, have been suggested as alternative specific pathogen-free (SPF) animal models that can be maintained in conventional facilities [30]. Among these, the Yucatan miniature piglet has been used as an experimental animal model for studies on influenza virus [31,32], human norovirus [33], and hepatitis E virus [34]. To the authors’ knowledge, no studies that use miniature piglets as an experimental animal model for PCV2 infection have been conducted so far.

In this study, we isolated a Korean field PCV2d strain (designated PCV2d/CBNU0324) from aborted fetal samples, and an experimental PCV2d-based inactivated vaccine was developed. The objective of this study was to investigate the use of an experimental infection model using miniature pigs infected with a Korean PCV2d isolate, and to evaluate the efficacy of an experimental PCV2d vaccine and a commercial PCV2b-based inactivated vaccine against PCV2d challenge in the same model.

## 2. Materials and Methods

### 2.1. Cells and Virus Isolation

The porcine kidney cell line PK-15, free of PCV1 contamination, was used for virus isolation. PK-15 cells were maintained at 37 °C with 5% CO_2_ in minimum essential medium alpha (MEM-α) (Invitrogen, Carlsbad, CA, USA) supplemented with 10% heat-inactivated fetal bovine serum (FBS), 2 mM L-glutamine, and antibiotic-antimycotic solution (Anti-anti).

**Figure 1:**
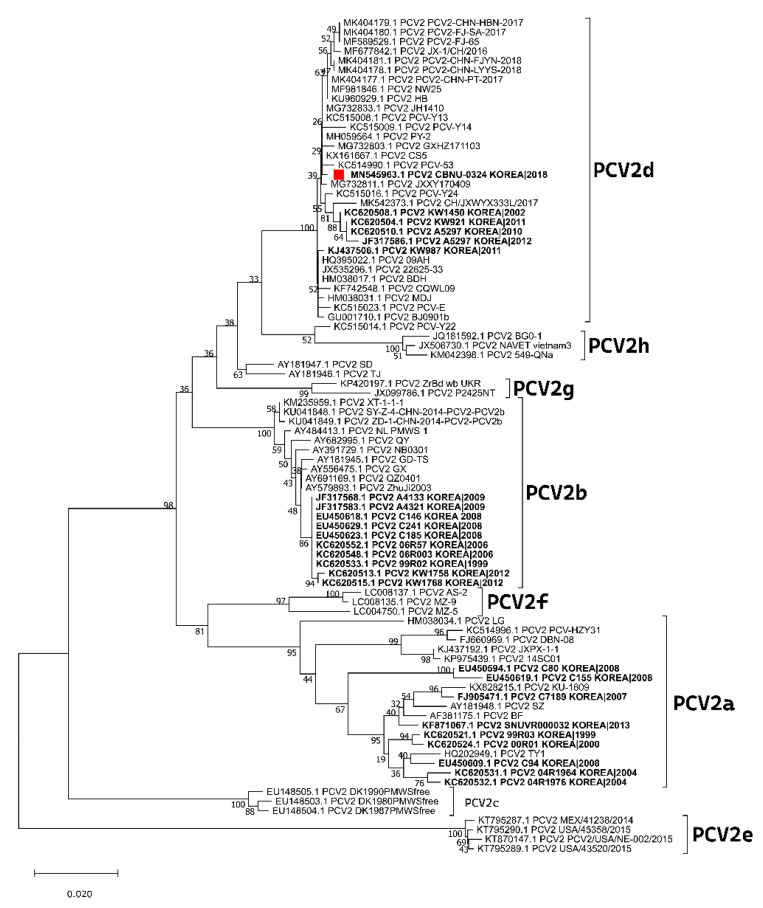
Maximum likelihood (ML) phylogenetic tree of the PCV2 ORF2 gene. The tree was generated using MEGA X software with 1000 bootstrap replicates. The PCV2d isolate used in this study (CBNU0324) is indicated with a red square symbol.

A clinical PCV2 isolate was recovered from the lungs of aborted fetuses that were submitted to the Jeonbuk National University Veterinary Diagnostic Center (JBNU-VDC) in 2017. Virus isolation was performed using a modified version of a previously described method [35]. The isolated sample was tested by differential PCR, and the ORF2 region was amplified with primer sets as previously described (Table 1) [17]. The isolated PCV2 strain was confirmed as genotype PCV2d-2 based on the phylogenetic analysis (Figure 1) and termed PCV2d/CBNU0324 (GenBank accession number MN545963). PCV2d/CBNU0324 was used for experimental vaccine development and challenge experiments in this study. The titer of PCV2 was estimated by determining the focus-forming units (FFUs) as previously described [36,37].

### 2.2. Vaccine Preparation

For experimental PCV2d-based vaccine production, PK-15 cells were cultured in alpha-minimum essential medium (α-MEM; Invitrogen, Carlsbad, CA) with 5% fetal bovine serum (Invitrogen) and antibiotic-antimycotic solution (100×; Invitrogen) and maintained at 37 °C in a CO_2_ incubator. PK-15 cells were prepared and subcultured with media for cell propagation. PCV2 was cultivated in prepared PK-15 cells for 18 h in the 37 °C CO_2_ incubator. The cultured fluid from the infected cell that was cultured for a certain period was discarded and incubated with 300 mM glucosamine for 15 min followed by washing with serum-free medium. After washing, the medium for cell propagation was added to the infected cell and cultured for 5 days, then the virus content was measured by fluorescent antibody technique. Each single dose of the vaccine was formulated to contain 10^6.5^ FFU/mL of PCV2. Formalin was added for the final concentration to be 0.2% and stirred with magnetic bar for 3 days to inactivate, then preserved at 4 °C. The bulk solution was then blended with a water-in-oil-in-water (W/O/W) multi-phase emulsion form of nanosome-type adjuvant (EUNGEN, Choong Ang Vaccine Laboratories Co., Ltd., Daejeon, Korea) at 10% (*w*/*w*) using the same method and composition as that of the commercialized vaccine (SuiShot^®^ Circo-ONE, Choong Ang Vaccine Laboratories Co., Ltd., Daejeon, Korea).

### 2.3. Animals

The 3-week-old SPF White Yucatan miniature piglets were purchased from Optipharm Inc. (Osong, Korea). All piglets tested negative for antigens and antibodies against porcine reproductive and respiratory syndrome virus (PRRSV), classical swine fever virus (CSFV), swine influenza virus (SIV), and PCV2 according to polymerase chain reaction (PCR) analysis and commercial ELISA tests.

### 2.4. Experimental Design

Two isolated animal experiments were conducted to assess the pathogenicity of the Korean PCV2d isolate (Experiment 1) and vaccine efficacy (Experiment 2) by using a miniature pig model. In experiment 1, a total of 6 piglets were randomly divided into two groups (3 piglets per group) and separately housed as a PCV2d infection group and a mock group. After acclimation for 2 days, the PCV2d infection group was inoculated with PCV2d/CBNU0324 (10^6.82^ FFU/mL) 1mL intramuscularly and 2 mL intranasally (1 mL in each nasal cavity). For the mock group, the piglets were inoculated with the same amount of PBS via the same route as that used in the infection group. Serum samples were collected at 0, 7, 10, 14, and 21 days post-inoculation (dpi), and samples of nasal and fecal swabs were collected at 0, 3, 5, 7, 10, 14, and 21 dpi. All the surviving animals were euthanized and necropsied to collect lungs, tonsils, submandibular lymph nodes (LNs), mediastinal LNs, tracheobronchial LNs, mesenteric LNs, inguinal LNs, ileums, colons, spleens, and livers for pathological assessment and viral load quantification.

In experiment 2, a total of 13 piglets were arbitrarily divided into three groups. The animals in group 1 (5 piglets) were intramuscularly administered a single 2 mL dose of a commercial PCV2b vaccine (SuiShot^®^ Circo-ONE, lot no: 318PCV02, Choong Ang Vaccine Laboratories Co., Ltd., Daejeon, South Korea) on the right side of the neck (PCV2b-vaccinated group, PCV2b-Vac). The animals in group 2 (5 piglets) were intramuscularly administered a single 2 mL dose of the experimental PCV2d vaccine (described above) on the right side of the neck (PCV2d-vaccinated group, PCV2d-Vac). The animals in group 3 (3 piglets) were intramuscularly administered the same dose of phosphate-buffered saline (PBS) and served as the nonvaccinated challenge group (Non-Vac). At 21 days post-vaccination (dpv), all piglets were challenged with PCV2d/CBNU0324 (10^6.82^ FFU/mL), 1 mL delivered intramuscularly and 2 mL delivered intranasally (1 mL in each nasal cavity). Two inoculation routes were used to ensure that all pigs became infected at the same time, as previously described [21]. All the surviving animals were euthanized and necropsied to collect the lungs, lymph nodes, and spleens at 21 days post-challenge (dpc) or 42 dpv for pathological assessment and viral load quantification. Serum samples were collected from all piglets in each group at 0, 7, 14, 21, 28, 35, and 42 dpv for antibody detection and quantification of serum viremia. Nasal swabs were collected after challenge at 21, 28, 35, and 42 dpv (0, 7, 14, and 21 dpc) for quantification of viral shedding.

Both animal experiments were performed at the Choong Ang Vaccine Laboratories Corporation (CAVAC) Animal Facility under the guidelines established by its Institutional Animal Care and Use Committee (approval number: 180212-03).

### 2.5. Clinical Evaluation

All piglets in the experiment were examined daily for clinical signs of weakness, lethargy, respiratory symptoms, anorexia, and lameness as previously described [38]. In experiment 1, the live weight of each piglet was measured at inoculation and every week after inoculation (0, 7, 14, and 21 dpi). In experiment 2, piglets were weighed at vaccination (0 dpv) and challenge (21 dpv or 0 dpc) and every week after challenge (7, 14, and 21 dpc). The average daily weight gain (ADWG, g/pig/day) was calculated as the difference between the starting and final weight divided by the stage duration and was recorded.

### 2.6. Serology and Measurement of Neutralizing Antibodies against PCV2

All serum samples were evaluated for PCV2 Cap-specific IgG antibodies with a PCV2 enzyme-linked immunosorbent assay (ELISA; VD pro PCV2 NC AB ELISA Kit; Median Diagnostics Inc., Chuncheon, Korea) in which sample-to-positive control (S/P) ratios less than 0.4 were considered negative results, according to the manufacturer’s instructions.

The levels of virus-neutralizing antibodies (NAs) were measured against PCV2d (CBNU0324) in the serum samples collected during experiment 2 by a fluorescence focus neutralization-based virus-neutralizing assay with PK-15 cells as previously described [37,39]. Sufficient levels of neutralizing antibodies were determined to be present when the number of infected cells decreased by 90% of the number of normal infected cells.

### 2.7. Quantitative Real-Time PCR

One gram of each collected tissue sample was homogenized with mechanical homogenizer (TissueRuptor, Qiagen, Korea Ltd., Seoul, Korea), mixed with 10 mL of PBS, and centrifuged at 2500 rpm for 10 min at 4 °C. Viral nucleic acids were extracted from the serum, nasal swab, and supernatant of homogenized tissue samples using MagMAX™ DNA Isolation Kits (Ambion, Austin, TX, USA) according to the manufacturer’s protocol. PCV2d DNA copy numbers were determined by amplification using PCV2 ORF2 gene-based real-time PCR using an AgPath-ID One-Step RT-PCR Kit (Applied Biosystems, Life Technologies, Carlsbad, CA, USA) with a primer and probe, as described in a previous study [40,41]. Samples with no signal by a cycle threshold (Ct) of 40 were considered negative for PCV2d [40,41,42].

### 2.8. Necropsy and Macroscopic Lesions

All piglets were necropsied to mainly observe gross lesions, enlarged lymph nodes, pulmonary collapse, and interstitial pneumonia. Gross lung lesion scores were estimated to reflect the amount of pneumonia in each lobe, as previously shown [21]. In short, ten points (five for the dorsal lobe and five for the ventral lobe) were assigned each to the right anterior lobe, right middle lobe, anterior part of the left anterior lobe, and caudal part of the left anterior lobe. The accessory lobe was assigned 5 points, and 27.5 points (15 for the dorsal and 12.5 for the ventral) were assigned to each of the right and left caudal lobes to reach a total of 100 points.

### 2.9. Microscopic Lesions and Immunohistochemistry

Microscopic lesions were “blindly” scored for tissue samples (lung, lymph node, and spleen) obtained from sacrificed piglets. Each sample was scored in terms of the severity of histological lesion on a 4-point scale, with 0 indicating the absence of histological lesion; 1, mild inflammation or mild inflammation with loss of overall cellularity; 2, moderate inflammation; and 3, severe inflammation with loss of lymphoid follicle structure or severe inflammation with replacement of follicles (Table 2).

Immunohistochemistry (IHC) of paraffin-embedded tissue sections was performed according to a previously described method [40]. Each specimen was scored semi-quantitatively in terms of the intensity of membrane immunostaining on a 4-point scale, with 0 indicating the absence of staining; 1, nonhomogeneous weak staining (<10% of the lymphoid follicles have cells with PCV2 antigen staining); 2, moderately positive staining (10–50% of the lymphoid follicles contain cells with PCV2 antigen staining); and 3, strongly positive staining (>50% of the lymphoid follicles contain cells with PCV2 antigen staining) (Table 2) [43,44].

### 2.10. Statistical Analysis

All continuous data (ADWG, PCV2 DNA copies, PCV2 antibody titers) were analyzed via two-way ANOVA followed by Tukey’s multiple-comparison test at each timepoint using GraphPad Prism, version 7.0 (GraphPad Software, San Diego, CA, USA). Discrete data (PCV2 viral load in tissues, macroscopic/microscopic lesions, and PCV2 antigen scores) were analyzed by one-way ANOVA followed by Tukey’s multiple-comparison test. *p*-values less than 0.05 were considered statistically significant.

## 3. Results

### 3.1. Experiment 1: Clinical Evaluation and Growth Performance

**Figure 2:**
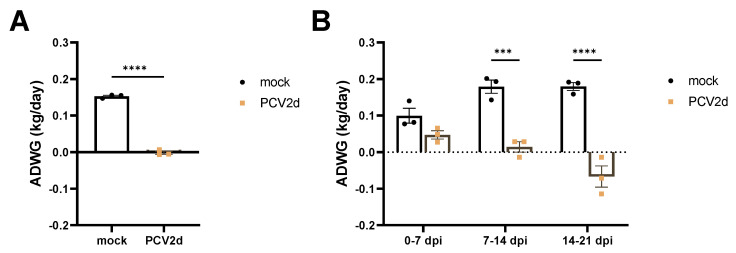
Growth performance of mock and PCV2d infection groups in the experimental infection/pathogenicity study (Experiment 1). The average daily weight gain (ADWG) was calculated during experimental periods: overall PCV2d infection (0–21 dpi, 21 days) (**A**), and each week after PCV2d infection (0–7 dpi, 7–14 dpi, and 14–21 dpi) (**B**). All data are presented as group means ± SEM. Statistically significant differences are indicated at *p* < 0.001 (***), and *p* < 0.0001 (****).

At 7 dpi, the PCV2d infection group showed mild diarrhea in two of three pigs. At 21 dpi, all three piglets in the PCV2d infection group showed cough concurrent with lethargy, weakness, and roughness of the hair (Figure S1), while those in the mock group showed no clinical symptoms. During the inoculation period, the ADWG observed in the PCV2d infection group was significantly (*p* < 0.0001) lower than that observed in the mock group (Figure 2A). In particular, a significantly (*p* < 0.001) delayed growth rate was observed after 7 dpi in the PCV2d infection group (Figure 2B).

### 3.2. Experiment 1: PCV2d Viremia, Viral Shedding, and PCV2-Specific IgG

**Figure 3:**
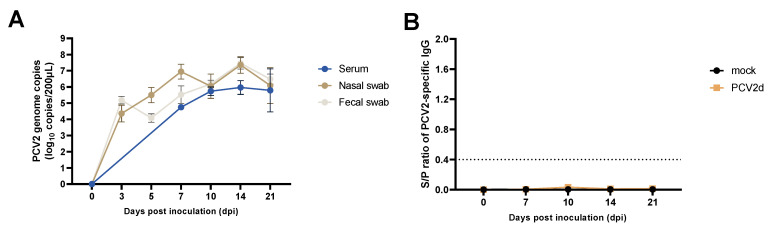
PCV2 genome copies and PCV2-specific IgG levels in the experimental infection/pathogenicity study (Experiment 1). Quantitative PCR analysis of PCV2 genome copies in the PCV2d infection group (**A**), and the levels of PCV2-specific antibodies in the mock and PCV2d infection groups (**B**). All data are presented as group means ± SEM.

PCV2d was not detected in the samples of serum or nasal or fecal swabs obtained from the mock group throughout the inoculation period. However, in the PCV2d infection group, a gradual increase in PCV2 genome copies was detected in all samples (Figure 3A). Increases in PCV2-specific IgG levels were not detected in any pigs in the PCV2d infection group or the mock group (Figure 3B).

### 3.3. Experiment 1: Viral Loads, Pathological Evaluation, and Detection of PCV2 Antigen in Tissues

**Figure 4:**
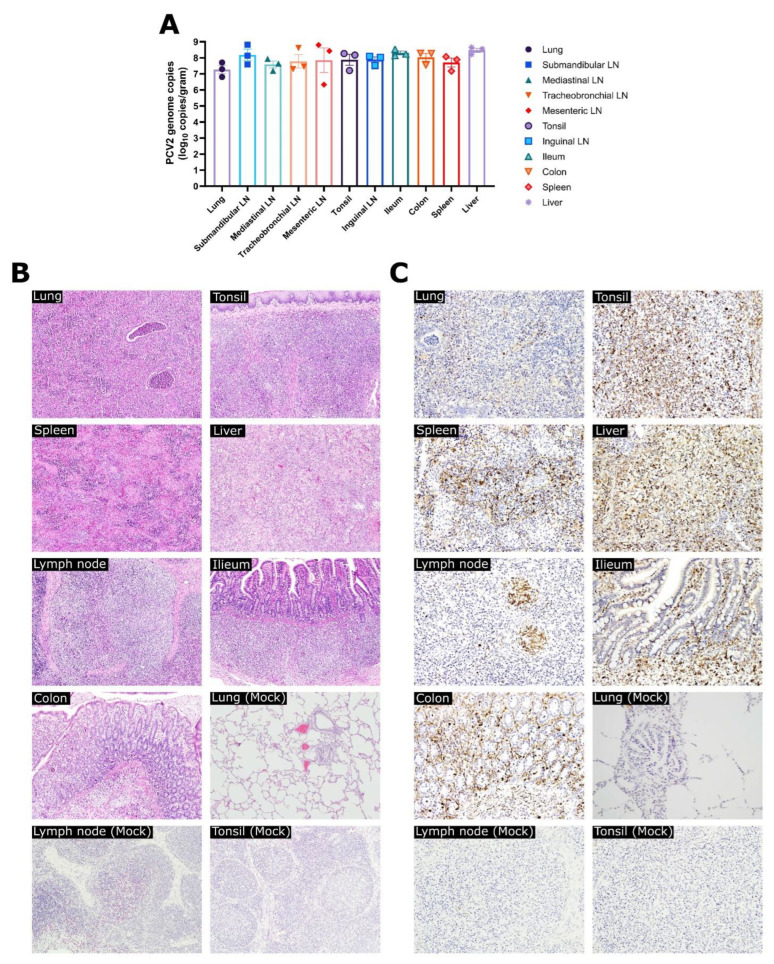
PCV2 genome copies, histological and immunohistochemical findings from PCV2d infection group in the experimental infection/pathogenicity study (Experiment 1). The levels of PCV2 genome copies in multiple organs obtained from the PCV2d infection group (**A**), and representative images of histological findings by H&E staining ((**B**), magnification: ×100) or immunohistochemistry staining ((**C**), magnification: ×200) within tissue samples obtained from the PCV2d infection group. The data of PCV2 genome copies are presented as group means ± SEM. Immunolabeling of PCV2 was observed as a dark brown signal.

No PCV2d genome copies were detected in any tissues obtained from the mock group. However, in the PCV2d infection group, high levels of PCV2 genome copies were observed in all collected tissues, with the highest levels observed in the liver (10^8.47^ copies/mL) and the lowest levels observed in the lung (10^7.27^ copies/mL) (Figure 4A). Patterns of interstitial pneumonia, lymph node enlargement and hemorrhagic findings, ascites, and spleen enlargement could be detected in the PCV2d infection group during necropsy. Under microscopy, only the PCV2d infection group showed various remarkable histological findings in tissues (Figure 4B and Table S1), including peribronchiolar and perivascular inflammatory cell infiltration and alveolar thickening within the lungs; histiocytic replacement of lymphoid follicles; and lymphoid depletion within the tonsil, spleen, and lymph nodes. In IHC examination, PCV2 antigens were detected in all sampled tissues obtained from the PCV2d infection group, while no PCV2 antigens were detected in the mock group (Figure 4C).

### 3.4. Experiment 2: Clinical Evaluation and Growth Performance

**Figure 5:**
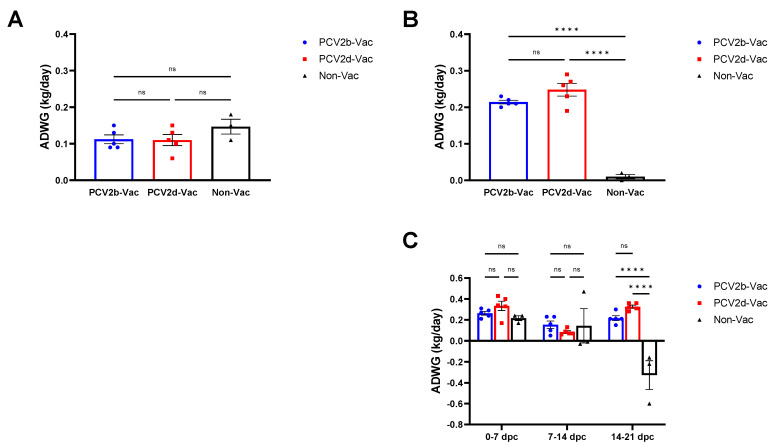
Growth performance of vaccinated and nonvaccinated pigs in the vaccine efficacy study (Experiment 2). The average daily weight gain (ADWG) was calculated during three experimental periods: vaccination to PCV2d challenge (0–21 dpv, 21 days) (**A**), PCV2d challenge to necropsy (0–21 dpc, 21 days) (**B**), and each week after PCV2d challenge (0–7 dpc, 7–14 dpc, and 14–21 dpc) (**C**). All data are presented as group means ± SEM. Statistically significant differences are indicated at *p* < 0.0001 (****).

Before challenge with PCV2d (0–21 dpv), the ADWGs of the PCV2b-Vac, PCV2d-Vac, and Non-Vac groups were 112, 112, and 147 g, respectively, with no significant difference (Figure 5A). From challenge to necropsy (0–21 dpc), the ADWGs of the PCV2b-Vac and PCV2d-Vac groups were 212 and 248 g, respectively, which were significantly (*p* < 0.0001) higher than that (10 g) of the Non-Vac group (Figure 5B). In particular, between 14 and 21 dpc, a significant (*p* < 0.0001) rapid decrease in ADWG in the nonvaccinated group was noted (Figure 5C).

### 3.5. Experiment 2: Serological Evaluation

**Figure 6:**
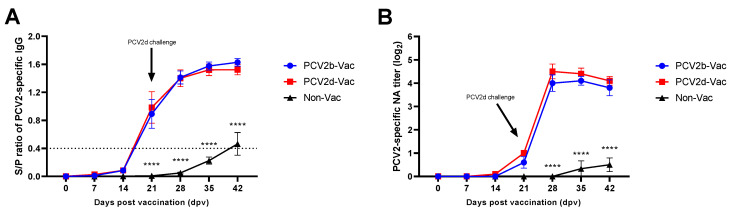
Antibody responses in pigs after immunization with different PCV2 vaccines in the vaccine efficacy study (Experiment 2). The levels of PCV2-specific IgG antibodies (**A**) and neutralizing antibodies (**B**) were detected at the indicated timepoints. All data are presented as group means ± SEM. Statistically significant differences between Non-Vac and vaccinated groups within the timepoint are indicated at *p* < 0.0001 (****).

In the vaccinated groups, a significant difference (*p* < 0.05) in the S/P ratio was observed compared with that of the nonvaccinated group, and four out of the five animals showed seroconversion at 21 dpv. PCV2-specific IgG titers in the vaccinated groups then rapidly increased after 28 dpv (7 dpc), reaching a significantly higher (*p* < 0.0001) antibody titer than that observed in the unvaccinated control group (Figure 6A).

Serum neutralizing antibodies (NAs) were observed at 21 dpv in both PCV2b- and PCV2d-vaccinated groups, and the neutralization responses elicited by both PCV2b and PCV2d vaccines were significantly (*p* < 0.0001) comparable in kinetics and magnitude to those observed in the Non-Vac group from 7 dpc (28 dpv) to 21 dpc (42 dpv) (Figure 6B).

### 3.6. Experiment 2: Viremia, Viral Shedding, and Viral Load in Tissues

**Figure 7:**
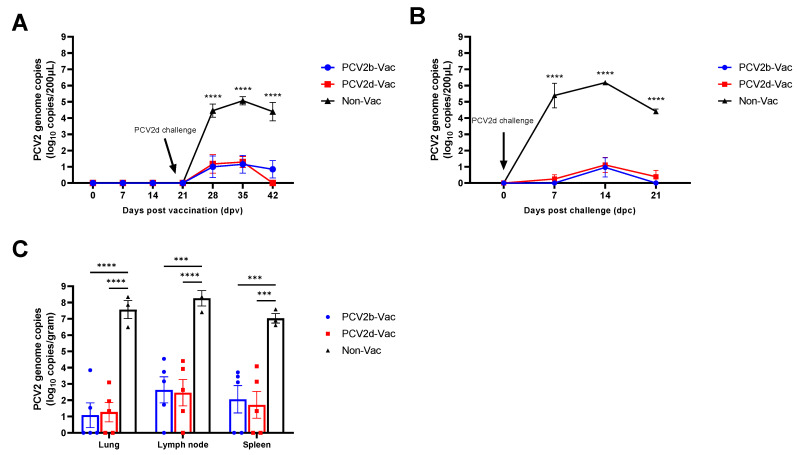
Quantitative PCR analysis of PCV2 genome copies in vaccinated and nonvaccinated pig serum (**A**), nasal swab samples (**B**), and tissue samples (**C**) in the vaccine efficacy study (Experiment 2). The viral DNA copies in the serum and nasal swab samples were detected by quantitative PCR at the indicated timepoints, and those in lung, lymph node, and spleen samples were detected at the time of necropsy. All data are presented as group means ± SEM. Statistically significant differences (*p* < 0.001, Tukey’s multiple-comparison test) between Non-Vac and vaccinated groups are indicated at *p* < 0.001 (***), and *p* < 0.0001 (****).

During the vaccination period, no viremia was observed in any of the groups. After challenge with PCV2d/CBNU0324, the Non-Vac group started to show significantly (*p* < 0.0001) higher levels of PCV2d viremia (10^4.45^ copies/mL) than the PCV2b-Vac (10^1.00^ copies/mL) or PCV2d-Vac (10^1.18^ copies/mL) groups at 28 dpv (7 dpc) (Figure 7A). A significantly higher level of PCV2d viremia (*p* < 0.0001) in the Non-Vac group was maintained until necropsy, while the viremia levels in the vaccinated groups remained low and gradually decreased.

Nasal virus shedding was evaluated during the challenge period (0–21 dpc). At 7 dpc (28 dpv), the Non-Vac group showed significantly (*p* < 0.0001) higher levels of viral shedding (10^5.39^ copies/mL), but almost no viral shedding was observed in the vaccinated groups (Figure 4B). A high level of viral shedding was maintained in the Non-Vac group until 21 dpc (42 dpv), while the vaccinated groups showed only a minimal level at 14 dpc (35 dpv) (Figure 7B).

All lymphoid tissues, including the spleens, lymph nodes, and lungs, of the unvaccinated control pigs showed a significantly (*p* < 0.0001) higher PCV2d load than those observed in the PCV2b- or PCV2d-vaccinated pigs (Figure 7C).

### 3.7. Experiment 2: Histopathological Lesions and PCV2 Antigens in Lymphoid Tissues

In two pigs in the PCV2b-Vac group, the lungs showed mild interstitial edema, while the lymph nodes showed mild enlargement or hemorrhage. In the PCV2d-Vac group, the lungs were normal. In the Non-Vac group, the lungs showed severe lung collapse, lung consolidations, a diffuse lobar pattern, and marked interstitial edema, which indicates interstitial pneumonia. For the lymph nodes and spleen, the vaccinated groups did not have any macroscopic lesions, while mild-to-intermediate enlargement and hemorrhage were found in the Non-Vac group. In terms of lung lesion score, the Non-Vac group showed a significantly higher score, 79.3 on average, than the vaccinated groups (*p* < 0.0001) (Table 3).

From lung, lymph node, and spleen tissue samples, lymphoid depletion and histiocytic-to-granulomatous inflammation were scored on histopathological examination. Compared to the Non-Vac group, the vaccinated groups showed significantly fewer histopathological lesions (*p* < 0.0001) in all tissue sample types examined (Table 3). Other histopathological findings, such as peribronchiolar and perivascular inflammatory cell infiltration, alveolar thickening, bronchiolitis, and pneumonia, were frequently detected in tissues of the Non-Vac pigs, in contrast to those of the vaccinated pigs (Table S2).

**Figure 8:**
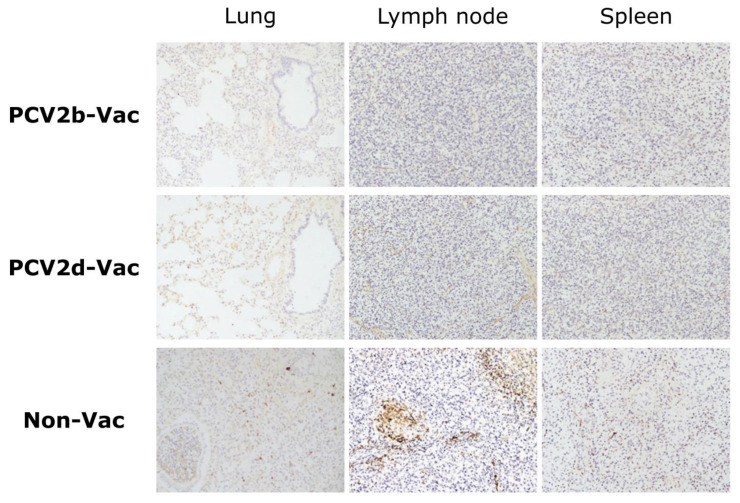
Representative images of immunohistochemistry assessment of PCV2 antigen in lung, lymph node, and spleen from PCV2b-Vac, PCV2d-Vac, and Non-Vac groups in the vaccine efficacy study (Experiment 2). Immunolabeling of PCV2 was observed as a dark brown signal. Magnification: ×400.

In IHC examination, PCV2 antigen was not found in any lymphoid tissues of either the PCV2b-Vac or PCV2d-Vac pigs, while the Non-Vac pigs exhibited significantly higher amounts of antigen in lymphoid tissues (Figure 8 and Table 3).

## 4. Discussion

Globally, vaccination has been identified as one of the most crucial and effective strategies to control PCV2-related diseases. As observed with the use of PCV2 vaccines in 2–3-week-old antibody-positive pigs, vaccination has had a major positive impact on decreasing PCVAD in the field [45]. Despite the widespread use of the PCV2 vaccine in domestic pigs, a genetic shift from PCV2b to PCV2d has raised an alarm about the possibility of a “leaky vaccine protection” situation. The use of vaccines can reduce only transmission, but it does not always induce sterile immunity, which may facilitate the evolution of the virus and the risk of vaccine failure [5]. Because of these concerns, studies regarding the cross-protective ability of several vaccines have been conducted, and the United States Department of Agriculture demanded that the results should be stated clearly when vaccines are launched [5,10,20,38].

Therefore, the objective of this study was to investigate the pathogenicity of the PCV2d genotype virus (currently the most prevalent genotype) and to evaluate inactivated and adjuvanted PCV2b- and PCV2d-based vaccines against PCV2d infection in a Yucatan miniature pig model. Most studies that conducted PCV2 vaccine and/or challenge experiments have used a conventional pig model [46,47,48,49,50]. However, the major challenges and disadvantages of using conventional pigs include difficulty in securing negative animals, poor test repeatability, and the difficulty of handling, thus prompting researchers to identify more convenient alternative animal models with characteristics of good genetic stability, small size, and susceptibility to infection [51]. Yucatan miniature pigs grow slowly and are smaller than other pig breeds when fully grown and have been found to have distinct traits, such as gentleness, intelligence, and relative lack of odor [52,53,54]. For these characteristics, Yucatan miniature pigs are used as laboratory animals worldwide, especially in studies of xenotransplantation [55], proteomics [56], and human pathogens [31,32,33,34]. However, the use of Yucatan miniature pig as an infection model of swine-specific pathogens including PCV2 has rarely been studied.

To investigate the impact of PCV2d single infection in a Yucatan miniature pig model, miniature pigs were inoculated with the PCV2d strain (CBNU0324) or treated as a mock group in experiment 1. While no changes were observed in the mock group throughout the experiment, significantly decreased ADWG (Figure 2) and high levels of viremia and nasal virus shedding were detected in the PCV2d infection group (Figure 3). Additionally, histological findings including bronchopneumonia, lymphoid depletion in lymphoid tissues, and inflammatory cell infiltration in multiple organs could only be detected in the PCV2d infection group (Figure 4 and Table S1). Although a direct comparison with conventional pigs was not conducted in this study, a single infection of PCV2 alone is known to rarely reproduce PCVAD [7,57,58,59], and other infectious or non-infectious agents need to be inoculated together to reproduce the clinical disease [43,60,61]. Thus, the obvious clinical outcomes and the reproduction of PCVAD with single infection of PCV2d that were observed in this study demonstrated that Yucatan miniature pigs could be used as an alternative experimental model of PCV2 challenge.

Experiment 2 was conducted to compare the protective efficacy between heterologous (PCV2b-based) and homologous (PCV2d-based) PCV2 vaccines against PCV2d challenge. Pigs that were nonvaccinated and singularly infected with the PCV2d strain (CBNU0324) exhibited similar clinical outcomes to those observed in the PCV2d infection group from experiment 1. Vaccinated groups (PCV2b-Vac and PCV2d-Vac), in contrast, showed significantly higher protective efficacy against PCV2d challenge than the Non-Vac group. After PCV2d challenge, both PCV2b and PCV2d vaccination significantly increased ADWG (Figure 5 and Figure 7) and dampened PCV2 virus replication and shedding, evidenced by significantly lower levels of PCV2 in serum samples and nasal swabs as compared with those in the Non-Vac group. Furthermore, the lymphoid PCV2d viral loads observed in the vaccinated groups were significantly lower than those observed in the Non-Vac group (Figure 7). In addition, stronger protection against PCV2 pathology and histopathology could be provided by PCV2b or PCV2d vaccination than by no vaccination (Figure 8 and Table 3).

Under the experimental conditions used with the SPF Yucatan miniature pig model in this study, PCV2 seroconversion was delayed, and very weak antibody responses were observed at 3 weeks after PCV2 challenge in the nonvaccinated group (Figure 6), which is similar to the results of previous studies [11,62]. In contrast, both the PCV2b- and PCV2d-vaccinated groups showed seroconversion at 21 dpv and maintained significantly higher ELISA and neutralizing antibody levels until necropsy than the nonvaccinated group (Figure 6). Therefore, the actively acquired antibody response induced by the vaccines successfully protected pigs, which was evidenced by a significantly lower PCV2 viremia level, an increased ADWG, and fewer PCVAD pathological lesions.

In this study, both the PCV2b and PCV2d vaccines were produced under almost the same conditions by using the same method for inactivation, the same adjuvant, and similar concentrations of vaccine antigen, and the vaccines successfully provided similar protective immunity in Yucatan miniature pigs against homologous/heterologous Korean PCV2d challenge. Most experimental and field PCV2 vaccine evaluations have been conducted with the commercially available PCV2a-based vaccine followed by challenge or natural exposure to PCV2a [24,46,47,48], PCV2b [24,47,49], or PCV2d [11,20,22,23,24,50]. In the case of PCV2d infection, which is currently the most prevalent in the field, experimental PCV2b-based or PCV2d-based vaccines have been evaluated by a few studies [11,45,63,64]. In the aforementioned studies, experimental PCV2 vaccines based on different genotypes have been shown to induce cross-protection against homologous/heterologous challenge with PCV2d, which is consistent with our study. Thus, as the outcome of vaccine efficacy against PCV2d challenge observed in the Yucatan miniature pigs used in this study successfully reproduced conditions similar to those observed in the conventional pig models, this study indicated that Yucatan miniature pigs could be an alternative model for vaccine protection experiments. Nevertheless, direct comparison between Yucatan miniature pigs and conventional pigs in PCV2 vaccine evaluation should be conducted in the future to identify whether these SPF minipigs could reflect vaccine efficacy in the field.

## 5. Conclusions

In conclusion, the results of this study indicate that the Yucatan miniature pig model could be an alternative experimental model of PCV2 challenge. In subsequent vaccine experiments, monovalent vaccinations with inactivated and adjuvanted PCV2b-based or PCV2d-based vaccines each provided similar and good levels of cross-protection against PCV2d challenge. Immunization with either vaccine effectively induced antibody responses, increased ADWG, and reduced levels of PCV2 viremia and microscopic lesions in Yucatan miniature pigs. Therefore, not only the PCV2d-based vaccine but also the PCV2b-based vaccine could help control PCVAD, in which highly prevalent PCV2d is mainly involved.

## Figures and Tables

**Table 1:** Primers used in this study.

Primer	Nucleotide Sequence (5′-3′)	Product Size	Purpose
F-PCV2C	GCT GGC TGA ACT TTT GAA AGT	1767 bp	PCV2 detection and sequencing
R-PCV2C	AAA TTT CTG ACA AAC GTT ACA		
PCV2ab 2NF	GGT TGG AAG TAA TCA ATA GTG GA	277 bp	PCV2a-specific
PCV2a 2NR	GGG GAA CCA ACA AAA TCT C		
PCV2ab 2NF	GGT TGG AAG TAA TCA ATA GTG GA	277 bp	PCV2b-specific
PCV2b 2NR	GGG GCT CAA ACC CCC GCT C		
PCV2d 2NF	GGT TGG AAG TAA TCG ATT GTC CT	343 bp	PCV2d-specific
PCV2d 2NR	TCA GAA CGC CCT CCT GGA AT		

**Table 2:** Scoring system for the severity of PCV2-associated histologic lesions and amount of PCV2 antigen.

Score	0 Points	1 Point	2 Points	3 Points
Lymphoid depletion	Not present	Mild, with loss of overall cellularity	Moderate	Severe, with loss of lymphoid follicle structure
Histiocytic-to-granulomatous inflammation	Not present	Mild	Moderate	Severe, with replacement of follicles
PCV2 antigen *	Not present	Less than 10%	10–50%	More than 50%

* Percentage of lymphoid follicles that have cells with staining for PCV2 antigen demonstrated by IHC.

**Table 3:** Comparison of the macroscopic lung lesion score, lymphoid lesion score, histiocytic-to-granulomatous inflammation score, and PCV2 antigen score between the vaccinated and nonvaccinated groups in the vaccine efficacy study (Experiment 2). All data are presented as group means ± SD.

		PCV2b-Vac	PCV2d-Vac	Non-Vac
Lung macroscopic lesion	Dorsal right lobe	2.4 ± 1.5 ^a^	1.2 ± 0.6 ^a^	18.3 ± 0.6 ^b^
	Dorsal left lobe	3.0 ± 1.0 ^a^	1.6 ± 0.5 ^a^	17.7 ± 1.5 ^b^
	Accessory lobe	1.1 ± 0.5	0.8 ± 0.3	2.7 ± 0.6
	Ventral right lobe	3.2 ± 0.8 ^a^	2.2 ± 0.4 ^a^	21.3 ± 2.1 ^b^
	Ventral left lobe	2.8 ± 0.8 ^a^	2.2 ± 1.1 ^a^	19.3 ± 3.5 ^b^
	Average of sum	12.5 ± 3.7 ^a^	8.0 ± 1.5 ^b^	79.3 ± 6.8 ^c^
Lymphoid depletion	Lung	0.2 ± 0.4 ^a^	0.0 ± 0.0 ^a^	2.0 ± 0.0 ^b^
	Lymph node	0.8 ± 0.4 ^a^	0.8 ± 0.4 ^a^	2.7 ± 0.6 ^b^
	Spleen	0.0 ± 0.0 ^a^	0.0 ± 0.0^a^	2.3 ± 0.6 ^b^
Histiocytic-to-granulomatous inflammation	Lung	0.8 ± 0.4 ^a^	0.6 ± 0.5 ^a^	2.7 ± 0.6 ^b^
	Lymph node	0.0 ± 0.0 ^a^	0.0 ± 0.0 ^a^	2.7 ± 0.6 ^b^
	Spleen	0.0 ± 0.0 ^a^	0.0 ± 0.0 ^a^	2.3 ± 0.6 ^b^
PCV2 antigen *	Lung	0.0 ± 0.0 ^a^	0.0 ± 0.0 ^a^	1.3 ± 0.6 ^b^
	Lymph node	0.0 ± 0.0 ^a^	0.0 ± 0.0 ^a^	2.0 ± 0.0 ^b^
	Spleen	0.0 ± 0.0 ^a^	0.0 ± 0.0 ^a^	1.3 ± 0.6 ^b^

* Percentage of lymphoid follicles that have cells with staining for PCV2 antigen demonstrated by IHC. Different superscripts (a, b, and c) indicate significant (*p* < 0.001) differences between groups.

## Data Availability

The data presented in this study are available in this article (and supplementary material).

## Supplementary Materials

The following supporting information can be downloaded at: https://www.mdpi.com/article/10.3390/vaccines10091469/s1. Figure S1: Postmortem findings from piglets of PCV2d infection group (n = 3) in the experimental infection/pathogenicity study (Experiment 1). Table S1: Histopathological findings in tissues of PCV2d infection group from experiment 1; Table S2: Histopathological findings in lungs, lymph nodes, and spleen of vaccinated and nonvaccinated groups from experiment 2.
