# Supplementary material for: Pathological Evaluation of Porcine Circovirus 2d (PCV2d) Strain and Comparative Evaluation of PCV2d and PCV2b Inactivated Vaccines against PCV2d Infection in a Specific Pathogen-Free (SPF) Yucatan Miniature Pig Model
Source: Vaccines (Basel). 2022 Sep 5;10(9):1469. doi: 10.3390/vaccines10091469 (PMC9501194; doi:10.3390/vaccines10091469)
Supplement: Supplementary file 1 [file vaccines-10-01469-s001.zip › Figure S1.pdf]

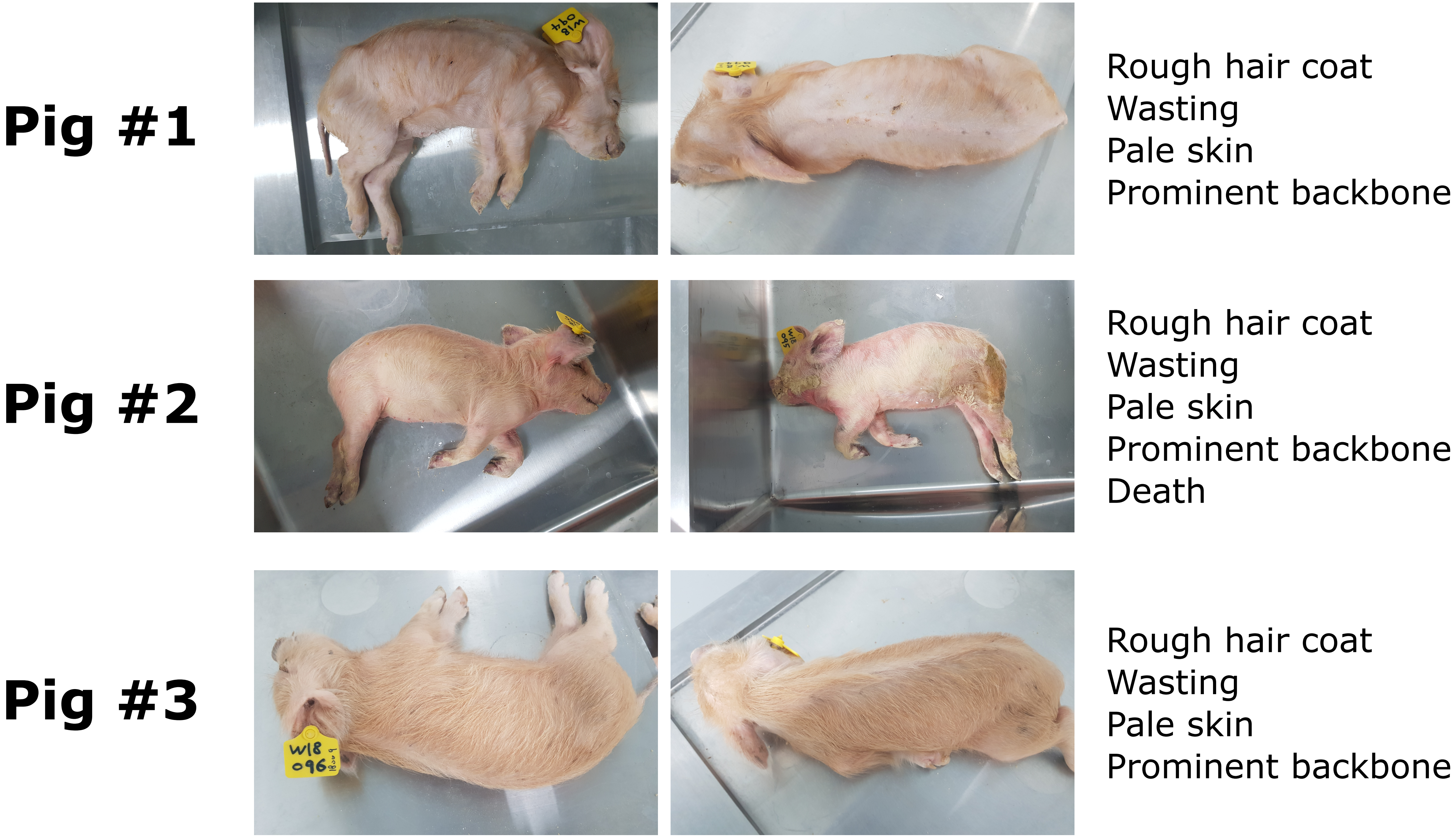

Supplementary Figure 1. Postmortem findings from piglets of PCV2d infection group (n=3) in the experimental infection/pathogenicity study (Experiment 1).
